# *lamaGOET*: an interface for quantum crystallography

**DOI:** 10.1107/S1600576721002545

**Published:** 2021-04-16

**Authors:** Lorraine A. Malaspina, Alessandro Genoni, Simon Grabowsky

**Affiliations:** a Universität Bern, Departement für Chemie, Biochemie und Pharmazie, Freiestrasse 3, 3012 Bern, Switzerland; b Universität Bremen, Fachbereich 2 – Biologie/Chemie, Institut für Anorganische Chemie und Kristallographie, Leobener Strasse 3, 28359 Bremen, Germany; c Université de Lorraine and CNRS, Laboratoire de Physique et Chimie Théoriques (LPCT), UMR CNRS 7019, 1 Boulevard Arago, 57078 Metz, France

**Keywords:** quantum crystallography, Hirshfeld atom refinement, X-ray constrained wavefunction fitting

## Abstract

In quantum crystallography, theoretical calculations and crystallographic refinements are closely intertwined. This means that the employed software must be able to perform both quantum-mechanical calculations and crystallographic least-squares refinements. So far, the program *Tonto* is the only one able to do that. The *lamaGOET* interface described herein deals with this issue since it interfaces dedicated quantum-chemical software (the widely used *Gaussian* package and the specialized *ELMOdb* program) with the refinement capabilities of *Tonto*. Three different flavours of quantum-crystallographic refinements of the dipetide glycyl-l-threonine dihydrate are presented to showcase the capabilities of *lamaGOET*: Hirshfeld atom refinement (HAR), HAR-ELMO, namely HAR coupled with extremely localized molecular orbitals, and X-ray constrained wavefunction fitting.

## Abstract 2

The program *lamaGOET* serves as an interface between quantum-mechanical and crystallographic refinement software to enhance the flexibility of the quantum-crystallographic methods Hirshfeld atom refinement and X-ray constrained wavefunction fitting.

## 1. Introduction   

An accurate determination of the electronic structure of a compound allows the derivation of many properties related, for example, to its reactivity or stability. One way of obtaining this information is through the theoretical calculation of a wavefunction for the compound under investigation. Wavefunctions are mathematical objects that intrinsically contain all the information of quantum-mechanical systems in specific pure states, most often the ground electronic state. Here, we are concerned with the electronic wavefunction, as the square of the electronic wavefunction is related to the electron density. Nowadays, with increasing computational power and the continuous development of sophisticated methods, many different software programs for calculation of wavefunctions are available, *e.g. Quantum ESPRESSO* (Giannozzi *et al.*, 2017 ▸), *Turbomole* (Furche *et al.*, 2014 ▸), *Crystal* (Dovesi *et al.*, 2018 ▸), *Gaussian* (Frisch *et al.*, 2016 ▸), *Orca* (Neese, 2012 ▸), *Tonto* (Jayatilaka & Grimwood, 2003 ▸) and many more.

A reconstruction of the electron density can also be achieved experimentally, *e.g.* from scattering experiments such as single-crystal X-ray diffraction. However, reconstructing the electron density of crystal structures always requires theoretical models to interpret the measured data, hence intrinsically connecting crystallography and quantum mechanics (Genoni *et al.*, 2018 ▸; Korlyukov & Nelyubina, 2019 ▸). The vast majority of crystal structure refinements use the independent atom model (IAM), where every atom is represented as a theoretically calculated spherical non-interacting averaged ground-state electron density (Compton, 1915 ▸; Sheldrick, 2008 ▸). This model ignores any deformation of electron density that is due to lone-pair regions, primary chemical bonding (covalent, metallic, ionic) and secondary interactions (*e.g.* hydrogen bonding, dipole–dipole interactions and London dispersion). There are electron-density models more accurate than the IAM that account for the nonsphericity of the atomic electron distributions (Koritsanszky & Coppens, 2001 ▸).

Multipole models (MMs) have been designed specifically to model chemical-bonding effects (Dawson, 1967 ▸; Kurki-Suonio, 1968 ▸; Hirshfeld, 1971 ▸; Stewart, 1976 ▸; Coppens, 2005 ▸). The most widely used MM variant is based on the Hansen–Coppens pseudoatom formalism (Hansen & Coppens, 1978 ▸; Coppens, 1997 ▸), where each atom is modelled by a superposition of radial and spherical harmonic functions. Atomic scattering factors are retrieved from a combination of tabulated spherical contributions and refined multipole parameters. This means that in multipole modelling both the molecular geometry, including atomic displacement parameters, and electron-density parameters are obtained by refinement against the measured structure factors. Alternatively, multipole parameters can be transferred from databanks (either constructed from theoretical calculations or averaged over experimental multipole refinements) and fixed during the refinement of positions and anisotropic displacement parameters (Dittrich *et al.*, 2005 ▸; Dadda *et al.*, 2012 ▸; Bąk *et al.*, 2011 ▸). By virtue of their construction, multipole databanks are suitable for the refinement of peptide and protein crystal structures with nonspherical atomic form factors (Jelsch *et al.*, 2000 ▸; Dittrich *et al.*, 2010 ▸).

Beyond multipole modelling, there are methods that make direct use of quantum-mechanical wavefunctions to model experimental diffraction data by taking into account atomic nonsphericity, and these are discussed in the following paragraphs. These methods belong to the emerging field of quantum crystallography (QCr) (Grabowsky *et al.*, 2017 ▸, 2020 ▸; Genoni *et al.*, 2018 ▸). The majority of QCr methods have to date been exclusively implemented and run in the software *Tonto* (Jayatilaka & Grimwood, 2003 ▸). The software *lamaGOET* presented in this work is a graphical user interface (GUI) for *Tonto* to make its full capability more easily accessible. Therefore, *lamaGOET* acts as an interface for the three quantum-crystallographic methods described below.

*Hirshfeld atom refinement (HAR)*. HAR (Jayatilaka & Dittrich, 2008 ▸; Capelli *et al.*, 2014 ▸) is an established method for modelling X-ray diffraction data with the help of nonspherical atomic scattering factors. In HAR, quantum-mechanical calculations are used to derive the theoretical electron density of the molecule under investigation. From this quantum-mechanical electron density, nonspherical atomic scattering factors, which are used in the refinement of the experimental data, are obtained using Hirshfeld’s stockholder partitioning of the electron density (Hirshfeld, 1977*a* ▸,*b* ▸). The following steps are performed during HAR:

(1) A single point energy computation provides an electron density distribution, using the current geometric parameters.

(2) The obtained electron density is then Hirshfeld partitioned into atomic electron-density functions (the Hirshfeld atoms), which are afterwards Fourier transformed to provide tailor-made nonspherical atomic scattering factors for the system under investigation.

(3) A least-squares refinement of positional and displacement parameters is carried out using the nonspherical scattering factors obtained in the previous step.

These steps are repeated until full convergence is achieved in energy and geometric parameters. The atomic scattering factors are purely theoretical, and only the atomic coordinates and the displacement parameters are refined against the experimental data. It has been shown that HAR is able to generate from X-ray data bond distances involving H atoms that are as accurate and precise as those obtained from neutron-diffraction studies (Woińska *et al.*, 2016 ▸; Fugel *et al.*, 2018 ▸; Sanjuan-Szklarz *et al.*, 2020 ▸), thus overcoming the limitations of IAM and MM in the determination of H-atom positions.

Through *lamaGOET*, HAR can be performed on the basis of wavefunctions calculated with the *Gaussian* software (Frisch *et al.*, 2016 ▸). In other words, *lamaGOET* allows interfacing *Tonto* and *Gaussian* directly. This gives access to quantum-mechanical methods otherwise not available, without detriment of any functionality in the original software *Tonto*.

*HAR-ELMO*. A limitation of HAR is the fact that it requires a Hartree–Fock (HF) or density-functional-theory (DFT) computation before each refinement step, so that it is computationally expensive. Hence, it cannot be readily applied to larger systems such as macromolecules or compounds containing heavy elements. However, Meyer & Genoni (2018 ▸) have recently constructed a library of extremely localized molecular orbitals (ELMOs). These molecular orbitals are strictly localized on small molecular units, *i.e.* atoms, bonds and functional groups. For this reason, they are easily transferable from molecule to molecule (Meyer, Guillot, Ruiz-Lopez & Genoni, 2016 ▸; Meyer, Guillot, Ruiz-Lopez, Jelsch & Genoni, 2016 ▸), allowing the quick reconstruction of wavefunctions and electron densities of proteins through instantaneous transfer of ELMOs from the databank with the software *ELMOdb* (Meyer & Genoni, 2018 ▸). At present, the databank includes ELMOs for all the naturally encoded amino acids. Therefore, for coordination compounds and corresponding ligands, ELMOs have to be calculated once before the transfer. It was recently demonstrated that the new HAR-ELMO method allows one to perform refinements that produce H-atom parameters as accurate and precise as those resulting from neutron diffraction or original HAR for small molecules (Malaspina *et al.*, 2019 ▸).

For HAR-ELMO applications, *lamaGOET* interfaces the *ELMOdb* software (Meyer & Genoni, 2018 ▸) with *Tonto* (Malaspina *et al.*, 2019 ▸). The *ELMOdb* software takes care of the rapid generation of wavefunctions from ELMO building blocks, *lamaGOET* transfers these wavefunctions to *Tonto*, and *Tonto* carries out the Hirshfeld atom partitioning and crystallographic least-squares refinement. Although other fragment approaches have recently been developed (Zheng *et al.*, 2020 ▸; Bergmann *et al.*, 2020 ▸), to the best of our knowledge, HAR-ELMO within *lamaGOET* is currently the only available method that has been used to refine a protein with quantum crystallographically derived nonspherical atomic scattering factors beyond multipole database techniques (Malaspina *et al.*, 2019 ▸).

*X-ray constrained wavefunction (XCW) fitting*. XCW fitting (Jayatilaka, 1998 ▸; Jayatilaka & Grimwood, 2001 ▸; Grimwood & Jayatilaka, 2001 ▸) allows for the fitting of molecular orbital coefficients to measured structure factors. Whereas HAR and HAR-ELMO as well as multipole database techniques derive nonspherical atomic form factors theoretically and refine only coordinates and displacement parameters, in the XCW strategy the electron density is refined via the fitting of molecular orbitals. This allows access to experimentally restrained wavefunctions.

A purely theoretical wavefunction is initially used as ansatz for the determination of the fitted wavefunction. In this procedure, instead of minimizing only the energy of the system under examination in a self-consistent field calculation, a new functional *L*[**c**] is minimized, which is the sum of the energy of the system *E*[**c**] and a term that represents the restraints given by the experimental X-ray diffraction data: 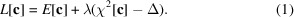


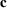
 is the matrix of the molecular orbital coefficients that are fitted to the experimental structure factors during the calculation, λ is an external multiplier that is manually adjusted during the computation and gives the strength of the experimental restraints, χ^2^ is a measure of the statistical agreement between experimental and theoretical structure factors, and Δ is the desired agreement between experimental and computed values. Therefore, in XCW fitting, experimental information is embedded into the theoretical wavefunction, in order to obtain the best possible description of the electron density.

During this procedure, all geometric parameters are unaffected. Therefore it is advisable to perform XCW fitting in the best possible derived geometry. The usage of XCW fitting after HAR is defined as X-ray wavefunction refinement (XWR) (Woińska *et al.*, 2017 ▸). *lamaGOET* can set up very specialized input files for XWR and XCW fitting procedures.

A large number of studies have shown that XCW fitting allows acquisition of reliable charge-density distributions for determination of material properties (Whitten *et al.*, 2006 ▸; Jayatilaka *et al.*, 2009 ▸; Hickstein *et al.*, 2013 ▸; Cole & Hickstein, 2013 ▸) as well as capturing polarization and crystal-field effects (Grabowsky *et al.*, 2020 ▸; Ernst *et al.*, 2020 ▸). Bytheway *et al.* (2007 ▸) and Bučinský *et al.* (2016 ▸) have also investigated theoretically what the detectability likelihood of electron correlation in diffraction data is, and Genoni *et al.* (2017 ▸) have proven that XCW fitting is in principle able to capture electron correlation to a certain extent.

In addition to the chemical example of glycyl-l-threonine dihydrate (Benabicha *et al.*, 2000 ▸) related to the three QCr methods described above, in this paper we also briefly discuss the scope of generating plots of various properties with *lamaGOET*, and how *lamaGOET* takes self-consistent Hirshfeld cluster charges from *Tonto* to perturb molecular wavefunctions in *Gaussian* geometry optimizations.

## 2. *lamaGOET*: platform, availability and use   

Traditionally, a myriad of different stand-alone utilities have been used by the crystallographic community, mostly in the Fortran programming language. However, more importantly, most crystallographic software can be run using a command-line interpreter. Therefore, interfacing different software can be easily achieved in command language. The *lamaGOET* interface started as a small bash script to perform a specific job, namely a HAR using *Gaussian* for the SCF calculations and *Tonto* for the refinements. Its utilities and functions rapidly increased, making it a tool for broad use in quantum crystallography. The latest version of *lamaGOET* is still written in bash, which makes it easy for users to read and understand the code. This also allows easy transferability across different operating systems. The script can be run on Linux and MacOS platforms using the native command-line interpreter. The prerequisites to run *lamaGOET* are usually default in any bash interpreter. These are *gawk*, *zenity* and *coreutils*. The increasing number of features and options led to the implementation of the graphical interface using *gtkdialog*, which is a GUI-creation utility that can be used with an arbitrary interpreter. By running the installation script provided with *lamaGOET*, all these dependencies are automatically installed, including *gtkdialog*. The *lamaGOET* script encourages code reuse and distribution and is subject to the GNU Public License. *lamaGOET* was written by LAM (lamaGOET = Lorraine A. Malaspina Gaussian Orca ELMO Tonto) (Malaspina, 2020 ▸) and can be obtained free of charge at http://www.tinyurl.com/lamaGOET (source code is also available at https://github.com/lomalaspina/lamaGOET).

**Figure 1:**
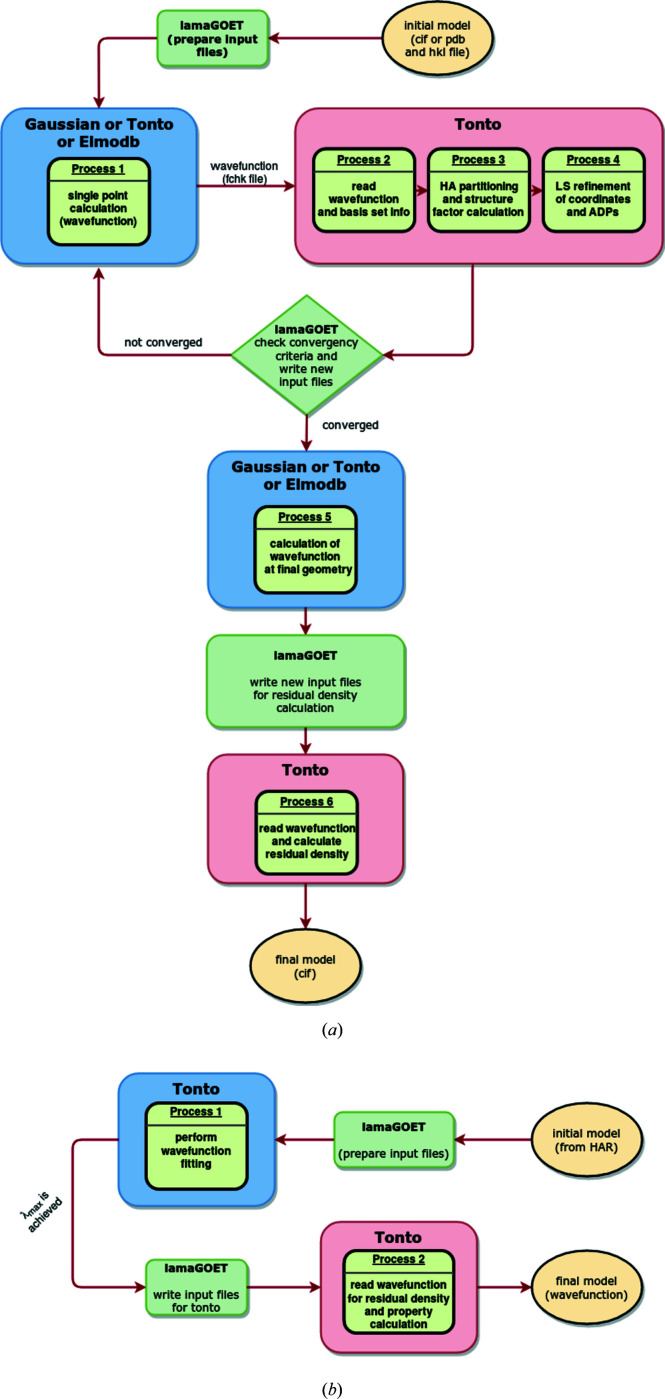
(*a*) Flowchart visualizing the procedure for HAR or HAR-ELMO controlled by *lamaGOET*, interfacing *Tonto* for the Hirshfeld stockholder partitioning and refinement with other quantum-mechanical software for the wavefunction calculation. (*b*) Procedure of the XCW fitting in *Tonto* controlled by *lamaGOET*.

Once the installation script has been run successfully, the GUI can be called by typing lamaGOET inside the chosen command-line interpreter from the folder where the result files are to be written. As input, a file which contains the initial geometry and crystal information of the structure is needed; this can be a CIF or a Protein Data Bank (PDB) file if the job uses the ELMO libraries. In addition, a reflection file in free format is needed as input for all quantum-crystallographic treatments. A flowchart visualizing the way in which *lamaGOET* enables HAR and HAR-ELMO by interfacing different quantum-mechanical software with *Tonto* is given in Fig. 1 ▸(*a*). Moreover, the procedure of an XCW fitting with *lamaGOET* is visualized in Fig. 1 ▸(*b*). Full explanations of all available functions and options to be chosen for the quantum-crystallographic examples discussed in Section 3 are given in the supporting information (Section 1).

There are a few special versions of *lamaGOET*. In Windows, it can be run using different available X servers or the GNU environment. It has been successfully tested on Windows platforms using the *MinGW*, *MobaXterm* and *Cygwin* tools. For use on computer clusters or supercomputers, two separate scripts are available for download. The first contains the graphical user interface (called *GUI-lamaGOET*), which should be placed on the user’s local machine. The second reads the inputs provided in the GUI and runs the script (*RUN-lamaGOET*). It should be placed on the computer cluster. Versions using the *Torque* (*PBS*) and the *YARN* (through spark.cmd) queuing systems are available at the github page https://github.com/lomalaspina/lamaGOET.

*Tonto* is available at https://github.com/dylan-jayatilaka/tonto. The github page provides step-by-step tutorials on how to install and compile *Tonto* for all different operating systems. We recommend to use gfortran-8 for the *Tonto* compilation on a Linux system.

*ELMOdb* is a stand-alone program (Meyer & Genoni, 2018 ▸) independent of *lamaGOET*. It allows the automatic transfer of ELMOs from the available ELMO databanks (Meyer & Genoni, 2018 ▸) to target polypeptide/protein structures. It requires a PDB file as input, which is analysed by the program one residue at a time. For each residue, *ELMOdb* processes every single fragment by retrieving the orbitals to be transferred and by defining the atomic triads that are necessary to define the matrices for the rotation/transfer of the ELMOs to the target structure (Meyer, Guillot, Ruiz-Lopez & Genoni, 2016 ▸; Meyer, Guillot, Ruiz-Lopez, Jelsch & Genoni, 2016 ▸; Meyer & Genoni, 2018 ▸). The current version of the ELMO libraries covers all the possible fragments for the 20 naturally encoded amino acids in all their possible protonation states and forms (N-terminal, C-terminal and non-terminal) and the water molecule. The stored ELMOs are available in different standard basis sets of quantum chemistry [6-31G, 6-31G(*d*,*p*), 6-311G, 6-311G(*d*,*p*) and cc-pVDZ]. In addition, *ELMOdb* has the option of reading customized ELMOs expanded on any basis set and corresponding to particular fragments, ligands or solvent molecules that may constitute the systems under examination. These tailor-made ELMOs must be preliminarily computed on appropriate model mol­ecules and then stored in a suitable directory where the program can retrieve them when necessary. *ELMOdb* finally provides an output file with general information on the performed computation, along with a binary file for the final rotated ELMOs, a binary file incorporating the associated ELMO one-electron density matrix, and a *Gaussian*-formatted checkpoint file that is used to perform subsequent analyses or calculations. The *ELMOdb* program and the ELMO libraries are currently available free of charge by sending a request to the main developer of the software (Alessandro.Genoni@univ-lorraine.fr) (Meyer & Genoni, 2018 ▸). In the course of a HAR-ELMO treatment (Fig. 1 ▸), *lamaGOET* reads the initial or iteratively refined geometry and passes it to *ELMOdb* for the automatic transfer and rotation of molecular orbitals, together with information on tailor-made residues (if present). *lamaGOET* then reads the formatted checkpoint file output from *ELMOdb* and passes it on to *Tonto* for the least-squares (LS) refinement.

The *lamaGOET* interface also facilitates the generation of grid files in *Gaussian* cube-file format within *Tonto* for different properties. These grid files are generated from the resulting binary wavefunction files written by *Tonto*. These binary files are generated through *lamaGOET* regardless of the software selected for the wavefunction-calculation step. At the moment, all *Tonto*-generated grid files will contain only atoms of the asymmetric unit which are within the unit-cell dimensions. Therefore, in many cases, pieces of molecules will be omitted for the calculation of cubes. To avoid this problem, *lamaGOET* offers the user the option to set the size, origin and orientation of the grid file manually. The next paragraph illustrates a short example of properties that can be calculated and plotted; further instructions on how to use this option can be found in the supporting information (at the end of Section 2 and in Figs. S5 and S9).

**Figure 2:**
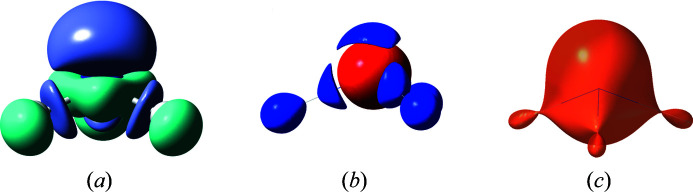
Different representations of bonding in ammonia; grid files defined with *lamaGOET* and calculated with *Tonto*. (*a*) Deformation density, isosurface at 0.016 e Å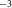, purple = positive, turquoise = negative. (*b*) Negative Laplacian of the electron density, isosurface at 35.7 e Å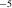, blue = positive, red = negative. (*c*) Becke88 exchange-correlation potential at 0.75 Hartree e^−1^. Graphics produced with the software *GaussView* (Dennington *et al.*, 2008 ▸).

In the ammonia crystal structure, only a third of the mol­ecule is symmetry independent. We performed HAR of ammonia based on experimental data taken from Boese *et al.* (1997 ▸). We used *Tonto* at the HF/def2-TZVP level of theory with the option to auto-complete the structure as described in the supporting information. The corresponding CIF is deposited with the Cambridge Structural Database (CSD) under CCDC deposition No. 1987830 and is also provided as supporting information to this article. Fig. 2 ▸ shows the related representations of the deformation density distribution, of the distribution of the negative Laplacian of the electron density and of the Becke88 exchange-correlation potential based on the Kohn–Sham orbitals calculated in the final HAR geometry. In the deformation density and the negative Laplacian, valence-shell charge concentrations (purple, blue) signify bonding and non-bonding effects, here the covalent N—H bonds and the nitrogen lone pair, respectively. The exchange-correlation potential is less structured, but it does show the presence of the nitrogen lone pair by a deviation from sphericity into the lone-pair direction.

Although unrelated to QCr, another option available inside the *lamaGOET* GUI is the possibility of setting up theoretical geometry optimizations of structures in *Gaussian* using a field of self-consistent Hirshfeld point charges within a defined cluster radius. Unlike the previous steps, this is a fully theoretical approach that allows the user to perform isolated-molecule optimizations with the influence of the environment. The idea is similar to that of the software *baerlauch* (Dittrich, Pfitzenreuter & Hübschle, 2012 ▸), where crystal structures are used to provide input files consisting of explicit clusters of molecules for *Gaussian* optimizations, but in *lamaGOET* the environment is considered implicitly via symmetry-generated cluster charges, not explicitly as in *baerlauch*. Geometry optimizations based on such simulations of the environment require significantly less computer power than a fully periodic calculation using the software described in the first paragraph of the *Introduction* [Sec sec1]. An example of this *lamaGOET* option is discussed in Section 3 of the supporting information.

## 3. Illustrative scientific example   

**Figure 3:**
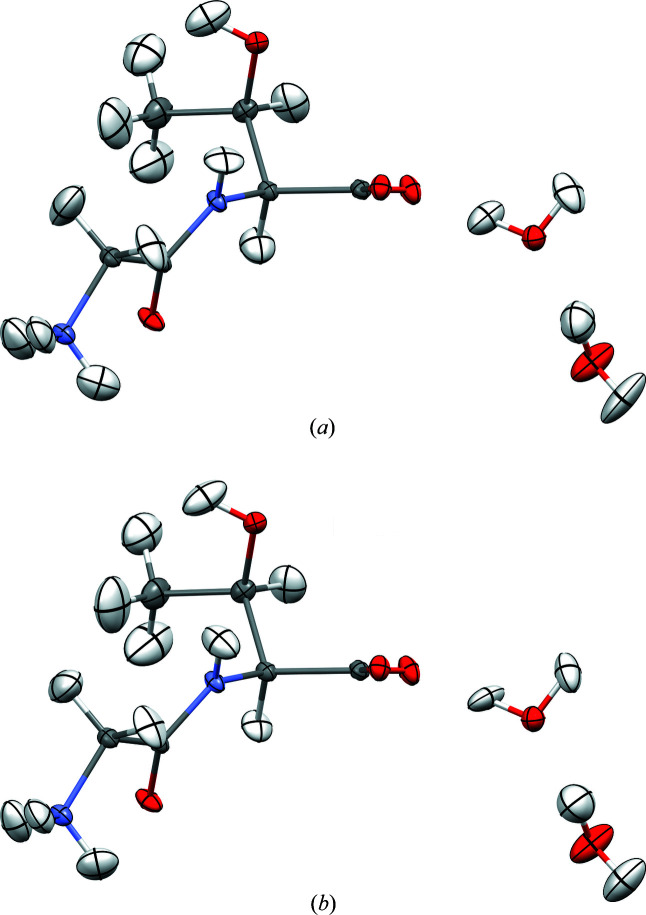
Structure of glycyl-l-threonine dihydrate obtained by (*a*) HAR-ELMO and (*b*) *Gaussian*-HAR. All ADPs are shown at the 50% probability level. Graphics produced with the software *Mercury* (Macrae *et al.*, 2020 ▸).

A high-resolution charge-density-quality data set of the dipeptide glycyl-l-threonine dihydrate was taken from the literature (Benabicha *et al.*, 2000 ▸). Some crystallographic and measurement details are repeated in Table 1 ▸. Two different HARs were performed on the available data: (i) HAR-ELMO with *lamaGOET*, *ELMOdb* and *Tonto* utilizing ELMOs expanded on the 6-311G(*d*,*p*) basis set, and (ii) a HAR with *lamaGOET*, *Gaussian* and *Tonto* (referred to as ‘*Gaussian*-HAR’) working with a wavefunction obtained at the B3PW91/def2-TZVP level of theory. The HAR-ELMO treatment is quick (13 min) and does not include any simulation of the crystal field. The *Gaussian*-HAR is more than seven times slower (94 min) but putatively more accurate, with a DFT functional only accessible via *Gaussian* (not available for ELMO generation or in *Tonto*) and a cluster of point charges around the asymmetric unit simulating the crystal-field effect. The refinement statistics are summarized in Table 2 ▸ and the molecular geometries with anisotropic displacement parameters (ADPs) for all atoms including H atoms are shown in Fig. 3 ▸. The corresponding CIFs are deposited with the CSD under deposition numbers 2027443, 2027444, 2027445 and 2027446 and are also provided as supporting information to this article.

**Figure 4:**
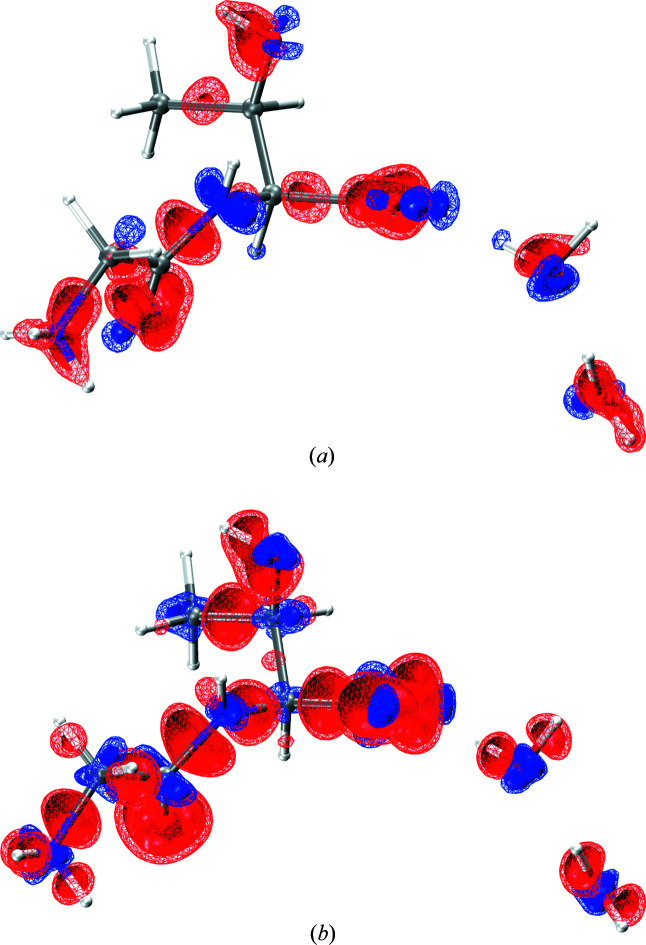
Difference electron density maps for (*a*) the transferred-ELMO wavefunction [6-311G(*d*,*p*) basis set] minus the HF/6-311G(*d*,*p*) wavefunction, and for (*b*) the B3PW91/def2-TZVP wavefunction surrounded by a cluster of point charges minus the HF/def2-TZVP wavefunction without a simulated crystal environment in glycyl-l-threonine dihydrate. Positive is blue, negative is red. The isovalue of the solid inner surface is 0.15 e Å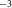 and that of the wireframe outer surface is 0.10 e Å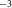. The images were generated with the software *VMD* (Humphrey *et al.*, 1996 ▸).

Fig. 4 ▸ visualizes the assumptions and approximations that are used in HAR-ELMO and *Gaussian*-HAR at the level of the electron density. Fig. 4 ▸(*a*) compares the transferred-ELMO wavefunction with a full Hartree–Fock wavefunction using the same basis set. The differences are systematic: only valence electron density is affected; bonding regions show negative difference density, and nitrogen and oxygen lone-pair regions positive difference density. This means that the extreme localization scheme used in HAR-ELMO underestimates the charge delocalization from lone pairs into bonding regions, which is especially pronounced for the resonance acting in the peptide and carboxylate functional groups.

Whereas Fig. 4 ▸(*a*) visualizes a methodological shortcoming in HAR-ELMO, Fig. 4 ▸(*b*) visualizes a methodological improvement in *Gaussian*-HAR when DFT and cluster charges are used instead of an isolated HF wavefunction. This means that Fig. 4 ▸(*b*) describes the combined effect of electron correlation (in the density-functional-theory approximation) plus polarization due to the crystal electric field (approximated by using Hirshfeld point charges) (Dittrich, Sze *et al.*, 2012 ▸; Kleemiss, Wieduwilt *et al.*, 2021 ▸). Overall, the combined correlation–polarization effect reduces electron density in the valence region and increases it in the atomic cores.

Despite the described differences in the underlying electron densities, the differences between the results of the fast but approximate HAR-ELMO and the slow but higher-level *Gaussian*-HAR are marginal. The figures of merit for both refinements in Table 2 ▸ are nearly the same. Only in the χ^2^ value does the *Gaussian*-HAR show a lower value, indicating a slightly better agreement between the model and the measured data. The freely refined hydrogen ADPs visualized in Fig. 3 ▸ appear to be physically meaningful in both models. The average C—H bond distances agree exactly between the two HAR models, but the O—H and N—H bonds are on average 0.01 Å longer in the *Gaussian*-HAR than in HAR-ELMO, and thus closer to reference values from neutron diffraction (Allen & Bruno, 2010 ▸). This is caused by the use of cluster charges in the *Gaussian*-HAR, whereas in HAR-ELMO the crystal environment is not accounted for (Fugel *et al.*, 2018 ▸). Notwithstanding this small advantage, the HAR-ELMO option in *lamaGOET* produces fast and reliable results for peptides and might be an option for future quantum-crystallographic refinement of protein crystal structures (Malaspina *et al.*, 2019 ▸).

Starting from the two slightly different geometries after HAR-ELMO and *Gaussian*-HAR treatments, two *lamaGOET*-mediated XCW fittings were performed at HF level, each of them without a surrounding cluster of charges. The reason for this choice is to test whether the electron correlation and polarization effects extracted through XCW fitting from measured structure factors are qualitatively and quantitatively comparable to those associated with the DFT and cluster charge approximations shown in Fig. 4 ▸, as well as to those reported in recent papers by Genoni *et al.* (2017 ▸) and Ernst *et al.* (2020 ▸).

**Figure 5:**
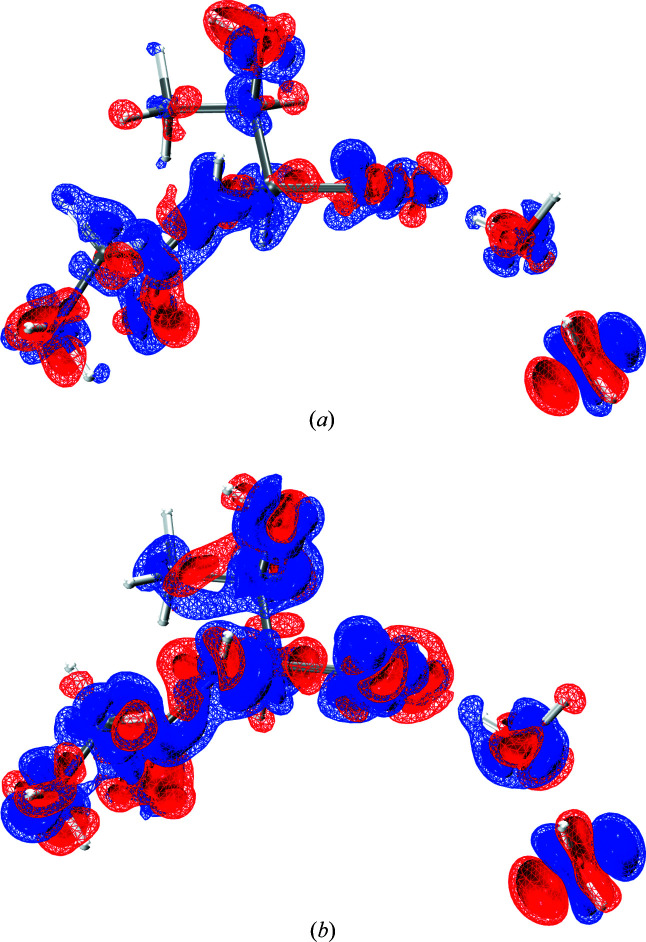
Difference electron density maps for (*a*) the XCW fitted wavefunction (λ = 3.0) minus the non-fitted wavefunction (λ = 0.0) at the level of theory HF/6-311G(*d*,*p*) and with the geometry from the HAR-ELMO treatment, and (*b*) the XCW fitted wavefunction (λ = 2.8) minus the non-fitted wavefunction (λ = 0.0) at the level of theory HF/def2-TZVP and with the geometry from the *Gaussian*-HAR treatment in glycyl-l-threonine dihydrate. Positive is blue, negative is red. The isovalue of the solid inner surface is 0.10 e Å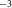 and that of the wireframe outer surface is 0.05 e Å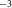. The images were generated with the software *VMD* (Humphrey *et al.*, 1996 ▸).

Fig. 5 ▸ shows that the XCW fitting effects on the electron density are qualitatively similar to the DFT and cluster-charge approximations visualized in Fig. 4 ▸, but they are smaller, as shown by the smaller isovalues used. There are two different reasons for this. (i) It has been shown that the XCW fitting effect depends strongly on the resolution of the data set and on the value of the external multiplier λ for fitting both electron correlation (Genoni *et al.*, 2017 ▸) and polarization (Ernst *et al.*, 2020 ▸). Here, a high-resolution data set is used (*d* = 0.44 Å) and a relatively small value of 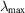 (2.8/3.0) was reached before convergence of the calculations ceased. (ii) The effect of electron correlation is overestimated by using a hybrid DFT functional (Medvedev *et al.*, 2017 ▸). In addition, the effect of polarization is overestimated by using self-consistent Hirshfeld charges (Kleemiss, Wieduwilt *et al.*, 2021 ▸). In summary, this means that the true effect of electron correlation and polarization on the electron density lies between Figs. 4 ▸(*b*) and 5 ▸.

## 4. Conclusions   

In this paper, we have demonstrated the usefulness and capabilities of the quantum-crystallographic interface *lamaGOET*. It facilitates Hirshfeld atom refinement, HAR-ELMO, X-ray constrained wavefunction fitting, the representation of properties on grids and the generation of a symmetry-generated crystallographic cluster of point charges for further theoretical calculations. At present, it is the only software that allows HAR-ELMO. *lamaGOET* is conceptually meant to be an interface for crystallographers who aim to work with *Tonto* for quantum-crystallographic applications and want to make the most of *Tonto*’s vast functionality, or even extend it with external quantum-mechanical software. *lamaGOET* will be maintained and expanded in this direction. In this sense, the development of *lamaGOET* is different from recent HAR developments in *Olex2* (Fugel *et al.*, 2018 ▸) and from connections between the tsc format of nonspherical atomic form factors (Midgley *et al.*, 2019 ▸) and HAR inside *NoSpherA2* (Kleemiss, Dolomanov *et al.*, 2021 ▸). These *NoSpherA2*-related developments make quantum-crystallographic refinements as simple and user friendly as possible and aim at a broad chemical audience, whereas *lamaGOET* remains a quantum-crystallographic tool centred around *Tonto*.

## Supplementary Material

Crystal structure: contains datablock(s) global, I. DOI: 10.1107/S1600576721002545/in5046sup1.cif

Structure factors: contains datablock(s) Gly-L-Thr. DOI: 10.1107/S1600576721002545/in5046Isup2.hkl

Supporting information pdf for publication. DOI: 10.1107/S1600576721002545/in5046sup3.pdf

Zip archive with all CIFs, structure factors, and checkcif pdfs. DOI: 10.1107/S1600576721002545/in5046sup4.zip

CCDC references: 1987830, 2027443, 2027444, 2027445, 2027446

## Figures and Tables

**Table 1:** Crystallographic and measurement details

Compound	Glycyl-L-threonine dihydrate
Chemical formula	C_6_H_12_N_2_O_4_·2H_2_O
Formula weight (g mol^−1^)	212.21
Crystal size (mm^3^)	0.300 × 0.300 × 0.150
Crystal habit	Rectangular prism
Crystal colour	Colourless
Temperature (K)	110 (5)
Wavelength (Å)	0.71068

Unit cell	
*a* (Å)	9.572 (3)
*b* (Å)	10.039 (3)
*c* (Å)	10.548 (3)
Volume (Å^3^)	1013.7 (5)
Crystal class/*Z*	Orthorhombic/4
Space group	*P*2_{1}2_{1}2_{1}

No. of reflections	15 903
*R*_int_	0.0233
Unique reflections	5417
Unique observed [*F*/σ(*F*) > 3]	4579
Reflections θ_max_ (°)	54.91 (*d* = 0.44 Å)

**Table 2:** Refinement statistics for glycyl-L-threonine dihydrate

	HAR		XCW fitting based on	
Refinement model	HAR-ELMO	*Gaussian*-HAR	HAR-ELMO	*Gaussian*-HAR
No. of parameters	271	271	1	1
No. of unique observations	4579	4579	4579	4579
*R* factor (obs)	0.029	0.029	0.027	0.026
*wR* factor (obs)	0.024	0.024	0.022	0.021
\chi^{2}	0.814	0.781	0.639	0.581
\lambda_{\rm max}	N/A	N/A	3.000	2.800
Residual density max (e Å^−3^)	0.312	0.315	0.315	0.340
Residual density min (e Å^−3^)	−0.319	−0.337	−0.285	−0.278
Residual density mean (e Å^−3^)	0.029	0.029	0.029	0.035
Time of the refinement (min)	13	94	N/A	N/A
CCDC deposition No.	2027445	2027443	2027446	2027444
