# Supplementary material for: lamaGOET: an interface for quantum crystallography
Source: J Appl Crystallogr. 2021 Apr 16;54(Pt 3):987–95. doi: 10.1107/S1600576721002545 (PMC8202027; doi:10.1107/S1600576721002545)
Supplement: Supplementary file 3 [file j-54-00987-sup3.pdf]

JOURNAL OF
APPLIED
CRYSTALLOGRAPHY

Volume 54 (2021)

Supporting information for article:

lamaGOET: an interface for quantum crystallography

Lorraine A. Malaspina, Alessandro Genoni and Simon Grabowsky

lamaGOET: An Interface for Quantum Crystallography (Supporting Information)

Lorraine A. Malaspina[a,b,*], **Alessandro Genoni**[c], **and Simon Grabowsky**[a,b,*]

[a]Universität Bern, Departement für Chemie, Biochemie und Pharmazie, Freiestrasse 3, 3012 Bern, Switzerland

[b]Universität Bremen, Institut für Anorganische Chemie und Kristallographie, Leobener Str. 3, 28359 Bremen, Germany

[c]Université de Lorraine & CNRS, Laboratoire LPCT, 1 Boulevard Arago, 57078, Metz, France

[*]lorraine.malaspina@dcb.unibe.ch, simon.grabowsky@dcb.unibe.ch

1 The Graphical User Interface lamaGOET

Figure S1 shows the main window of the *lamaGOET* GUI where the different input options are highlighted in red. They are explained in the following:

1. Option to select different QM software packages available for the single-point energy calculation. Once one of these is selected, all the other options inside the GUI will automatically be enabled/disabled according to their availability for use in conjunction with the selected QM software.

2. If *ELMOdb* is selected, the *ELMOdb* program is used. This checkbox also allows to ask for an interface to use the program *GamessUK* for the calculation of overlap integrals between basis functions to be used in *ELMOdb* (if necessary).

3. Executable name (if a global link is set) or full path to the *Tonto* executable. *Tonto* can be downloaded free of charge at https://github.com/dylan-jayatilaka/tonto.

4. Executable name (if a global link is set) or full path to the software selected in option 1 (if different than *Tonto*).

5. *GamessUK* executable name if item 2 is selected.

6. Path to the ELMO libraries (if *ELMOdb* is selected in 1).

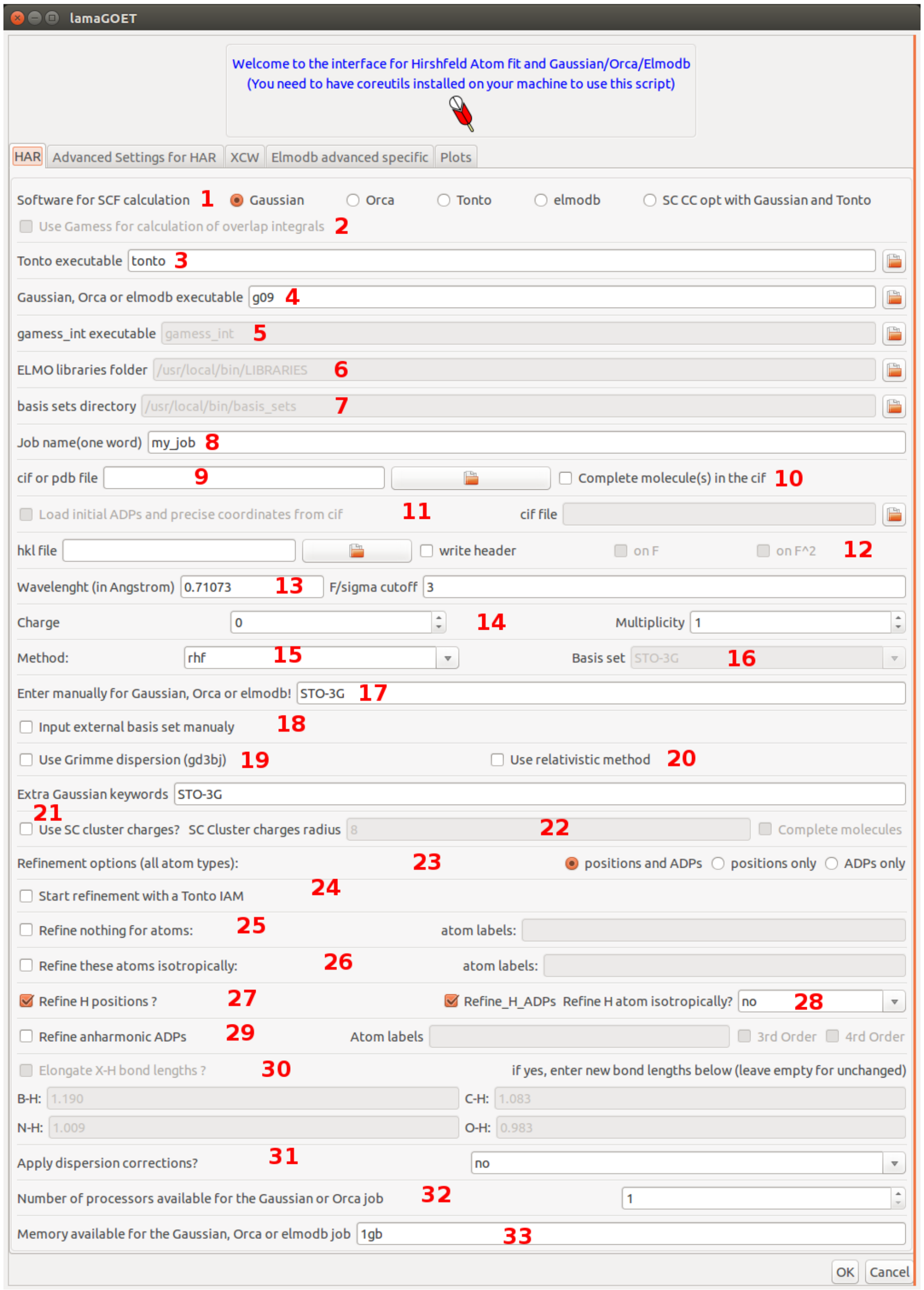

Figure S1. *lamaGOET* graphical interface main window with all input options highlighted by red numbers that are explained in the text.

7. Path to the *Tonto* basis-set folder if *Tonto* is selected for the wavefunction calculation step, or the *ELMOdb* basis-set folder for the *ELMOdb* option. For all other cases *Tonto* will read the basis-set information out of the respective file (e.g. fchk) obtained in the wavefunction calculation step.

8. Job name which will be the new data block name in the resulting CIF and also in every file for the current refinement.

9. Input CIF that contains the structure to be refined. In the case of *ELMOdb*, this is a PDB file.

10. Option to be used in case of Z' $<$ 1 to automatically complete the molecule from the asymmetric unit. Do not use if you have a network compound!

11. Option to provide initial ADP information in case *ELMOdb* is selected. This is not a compulsory step, but usually providing initial ADPs might help to reach convergence in fewer steps in a HAR-ELMO refinement.

12. Selection of the reflection file and the option to automatically include the header required by *Tonto*. If the header is already contained in the reflection file, the software will automatically ignore the request.

13. Wavelength of the experiment (by default set to Mo$\kappa\alpha$), and the desired F/σ cutoff.

14. Charge and multiplicity of the input geometry.

15. Selection of QM methods available simultaneously in *Tonto* and *Gaussian*. Any other method not displayed in the drop-down menu can be entered manually by typing it in the same input box.

16. All basis sets available in *Tonto* are listed in a drop-down menu. If a different basis set is required for the single-point calculations in *Gaussian*, it can be entered manually in field 17.

17. *Gaussian* or *ELMOdb* basis-set name.

18. Option to provide the basis set for *Gaussian* explicitly. If selected, a pop-up window will appear where the basis-set information should be entered. The expected format is explained with an example and it should follow the same format as provided in the EMSL basis-set exchange website (https://www.basissetexchange.org/EMSL) for *Gaussian*. One can usually copy and paste the information from the EMSL website directly into the pop-up window. It sometimes seems as if

some special characters such as the carriage return are wrong in the entered text, but in fact they are not. The *lamaGOET* program takes care of the special characters.

19. Option to use Grimme's D3BJ dispersion [1] in the wavefunction calculation if *Gaussian* is selected under 1.

20. Option to use relativistic methods (Douglas-Kroll-Hess [2, 3]) inside *Gaussian* if *Gaussian* is selected under 1.

21. Option to use self-consistent cluster charges during the calculation of the wavefunction. The addition of the effect of neighboring molecules through the self-consistent cluster charges is relevant to simulate the crystal field, especially for strong hydrogen bonds. Point charges are calculated by *Tonto* from Hirshfeld partitioning. In this paper, only monopoles were used in Gaussian-HAR, unlike in regular Tonto HARs where monopoles and dipoles (mimicked by point charges of opposite sign at 0.001 a.u. distance to each other) are used. In the future, Tonto output will include these dipoles and can then also be used for Gaussian-HARs.

    If the structure is not a network compound, it is advisable to always complete the molecule before the cluster generation. This option is not available if *ELMOdb* is selected because the ELMOs that are stored in the ELMO libraries are transferred in order to reconstruct the wavefunctions of isolated molecules/systems (e.g., the asymmetric unit) regardless of the crystal environment.

22. Radius of sphere for the calculation of cluster charges and options to complete the molecules at the edge of the cluster. Do not select the option to complete the molecules if you are working with network compounds.

23. Refinement options for positions and ADPs for all atoms.

24. Option to perform an IAM pre-refinement before HAR.

25. Option to enter the atom labels (separated by space) of the atoms that will be kept frozen in the refinement.

26. Option to refine the following atom labels using isotropic behavior only.

27. Option to refine or not to refine hydrogen atom positions.

28. Option to refine hydrogen atoms isotropically or anisotropically.

29. Option to refine higher-order ADPs for certain atom labels through 3rd and/or 4th order Gram Charlier coefficients [4].

30. Option to elongate X-H distances for all hydrogen atoms in the structure (regardless of the hybridization state of the atom to which it is bonded). This option is not available if the option to perform pre-IAM is selected.

31. Option to add anomalous dispersion corrections into the calculated structure factors. If activated, a pop-up window will appear to enter f' and f" coefficients for each element that should be included in the anomalous dispersion correction. It is not required to enter these values for every element in the structure.

32. Option for the number of cpus to be used for the wavefunction calculation. If a parallel *Tonto* version (message passing interface (mpi)) is installed, this will also be used for the *Tonto* step.

33. Memory available for the *Gaussian* or *ELMOdb* (old version) jobs. The memory should contain the units mb or gb for *Gaussian*, but for *ELMOdb* (old version) only mb are available, and the number should be entered without units (there is an upper limit of 5000 for *ELMOdb* (old version)).

The second tab named "Advanced Settings for HAR" allows to change default values such as the convergence criteria inside *Tonto* (Figure S2). By default, the convergence criterion is 0.01 for the maximum shift of any parameters over its standard uncertainty inside the least-squares (L.S.) refinement. One can also limit the number of iterations inside each L.S. cycle. Besides this, *lamaGOET* also has an internal upper limit of 50 interations (wavefunction calculation plus L.S. refinement). If using DFT methods within a refinement that is strictly based on *Tonto* only, options within this tab can be used to change the Becke grid accuracy and pruning scheme [5]. (For more information regarding the accuracy and pruning scheme available please refer to the *Tonto* github page).

The third tab (Figure S3) labelled "XCW" contains options to run XCW fittings (exclusively with *Tonto*) and X-ray Wavefunction Refinement (HAR + XCW: where HAR can be run with all different software available, but the XCW part exclusively with *Tonto*) following the level of theory defined in this tab.

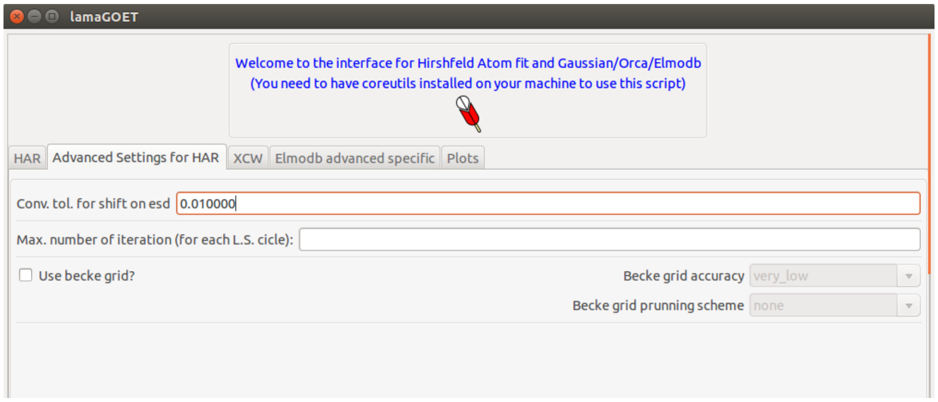

Figure S2. *lamaGOET* GUI. Second tab: Advanced settings for HAR.

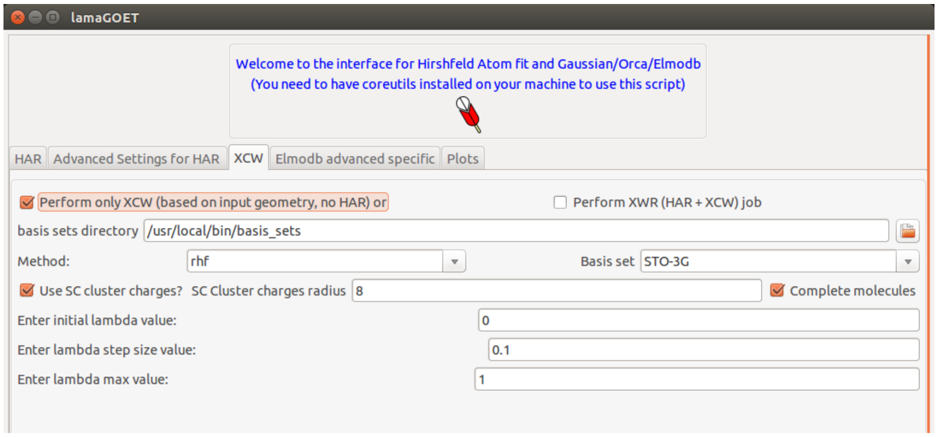

Figure S3. *lamaGOET* GUI. Third tab: XCW.

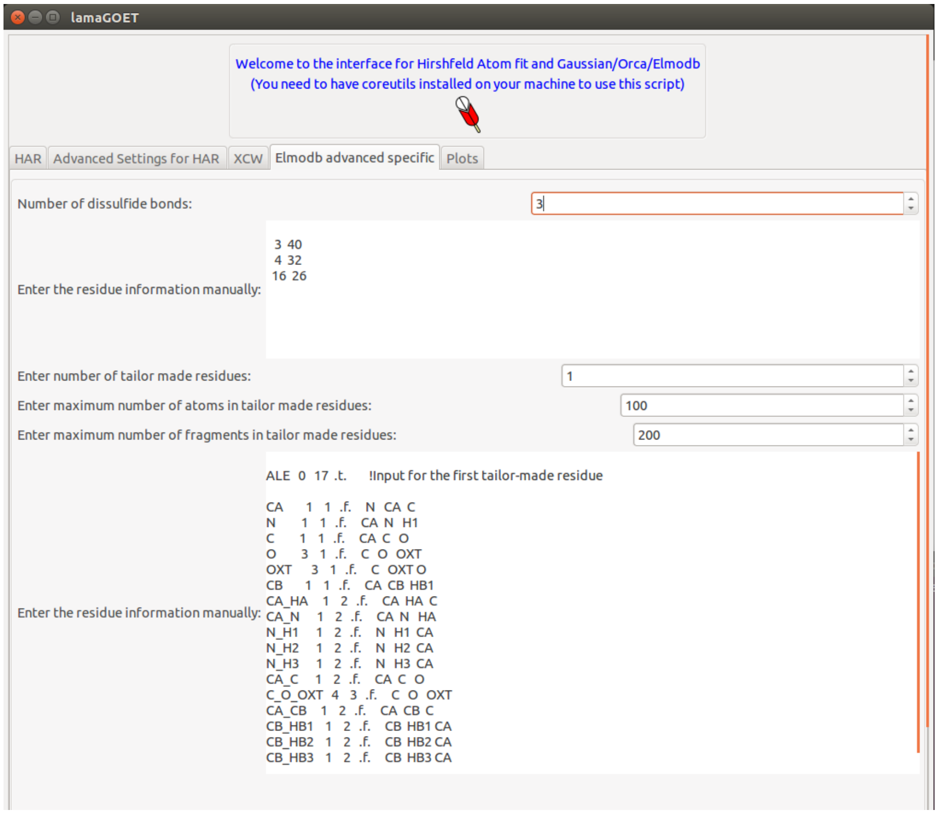

Figure S4. *lamaGOET* GUI. Fourth tab: ELMOdb advanced specific.

The fourth tab (Figure S4) corresponds to advanced options when using the *ELMOdb* program. In this tab, the number of disulfide bonds (if any) must be entered, followed by the number of the residues between which the disulfide bonds occur (one line per disulfide bond, with residue numbers separated by at least one space as shown in the pre-filled example for crambin, where three disulfide bonds can be found between residues 3 and 40, 4 and 32, and 16 and 26). If tailor-made ELMOs are used (namely ELMOs not provided within the AMINO-ACIDS folder within the ELMO libraries), the number or tailor-made residues should be also entered in this tab. The maximum number of atoms and fragments for the tailor-made residues should also be provided. Finally, in the last box of the tab, for each customized residue, the user should indicate the corresponding name (e.g., ALE in the example shown in Figure S4), along with other information about the constituting fragments on which the ELMOs are localized.

The fifth and last tab labelled “Plots” (Figure S5) allows to plot several properties using the wavefunctions obtained from the performed calculations. All plots generated here will be in a *Gaussian* cube format which can be opened with different visualization software packages.

All the information provided here can also be seen as a help text inside the GUI by hovering the mouse

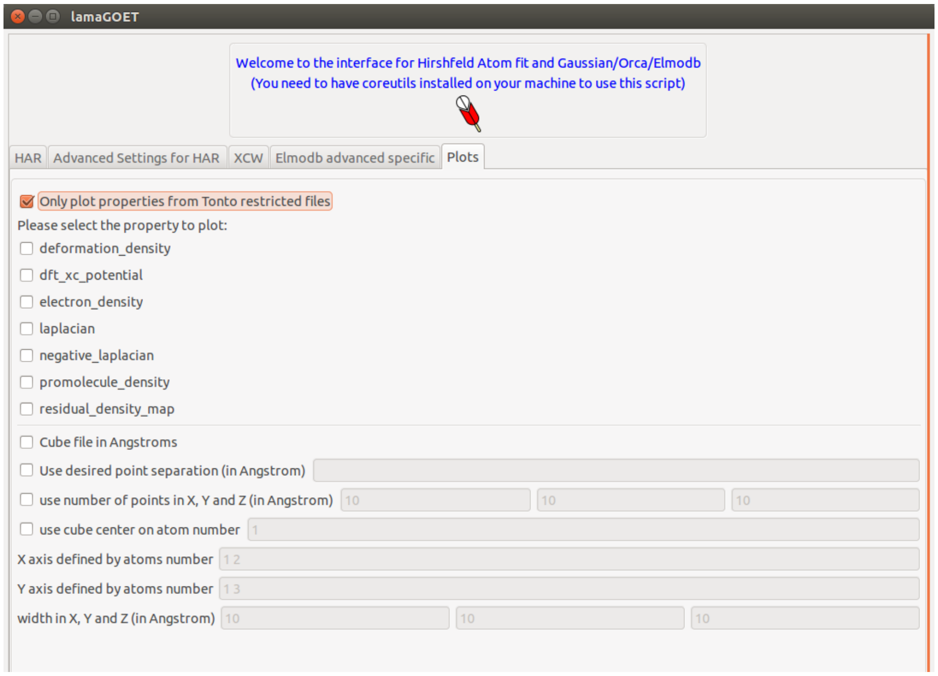

Figure S5. *lamaGOET* GUI. Fifth tab: Plots.

over the respective field.

When the OK button is pressed to start a refinement, a text file named *job_options.txt* will be written in the work directory assigning the values of the variables used inside the program according to the information provided. If the GUI is started in a folder that already contains a *job_options.txt* file, the GUI will be pre-filled with the values stored in the *job_options.txt* file.

2 Example input for lamaGOET jobs

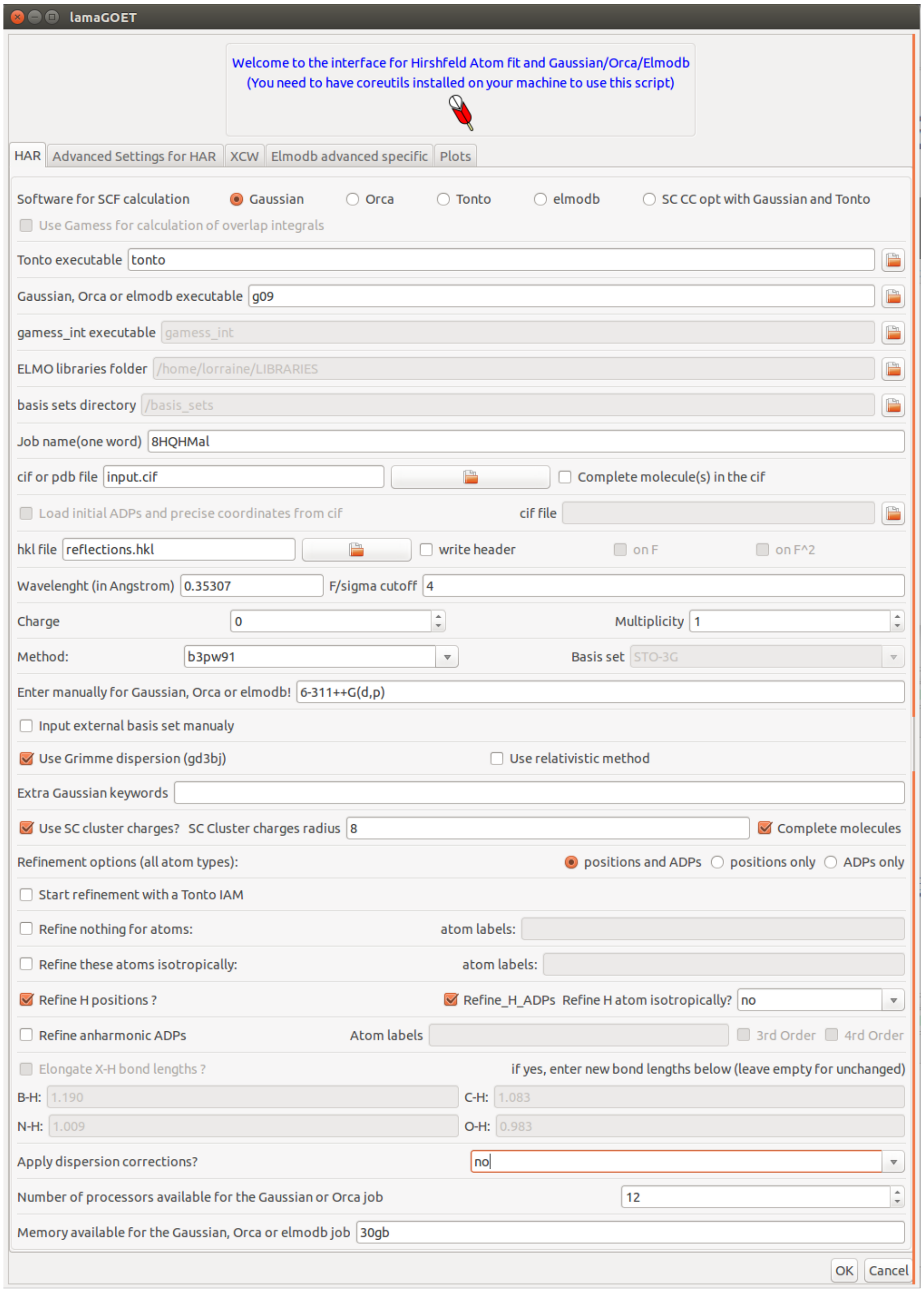

Figure S6. *lamaGOET* GUI with options for a *Gaussian*-HAR.

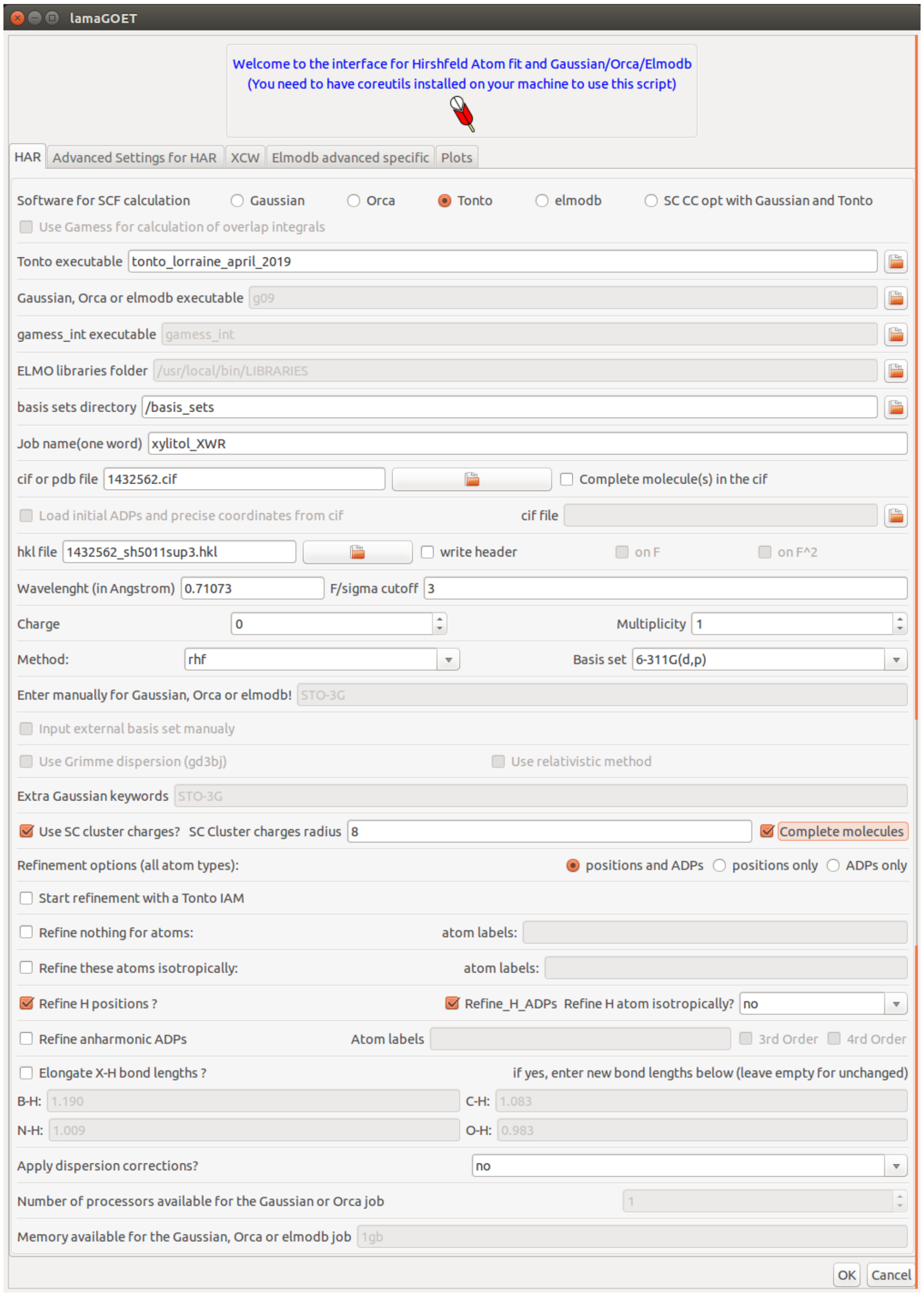

Figure S7. *lamaGOET* GUI for a regular HAR using *Tonto*.

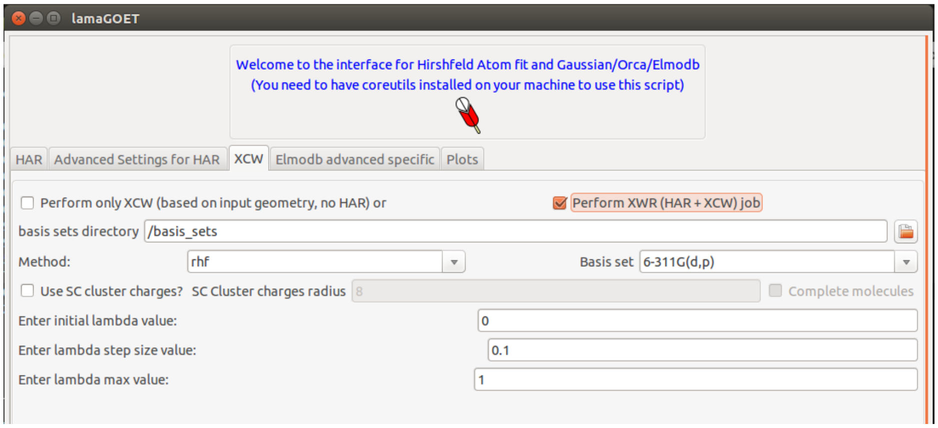

Figure S8. *lamaGOET* GUI XCW tab showing the options for a XCW fitting using *Tonto*.

When a part of the asymmetric unit is outside the unit cell, a box different from the asymmetric unit or unit cell needs to be specified to calculate properties with *Tonto*. For the specification of the center and axes of the new box (cube), *Tonto* requires the atom number (not the label) according to the order in which it appears in the input file.

Figure S9 shows the input in *lamaGOET* to generate a cube file of the deformation density for a random molecule, where the center was placed at atom number 8. The x axis was defined pointing from atom 9 to atom 10. The y axis was defined as pointing from atom 7 to atom 2, and the z axis is automatically generated within *Tonto* as the vector product of x and y. The length of each axis was also specified (15 Å for x, and 10 Å for y and z) as well as the desired separation of points of 0.1 Å. Another option would be to specify the number of points along each axis instead of the desired separation.

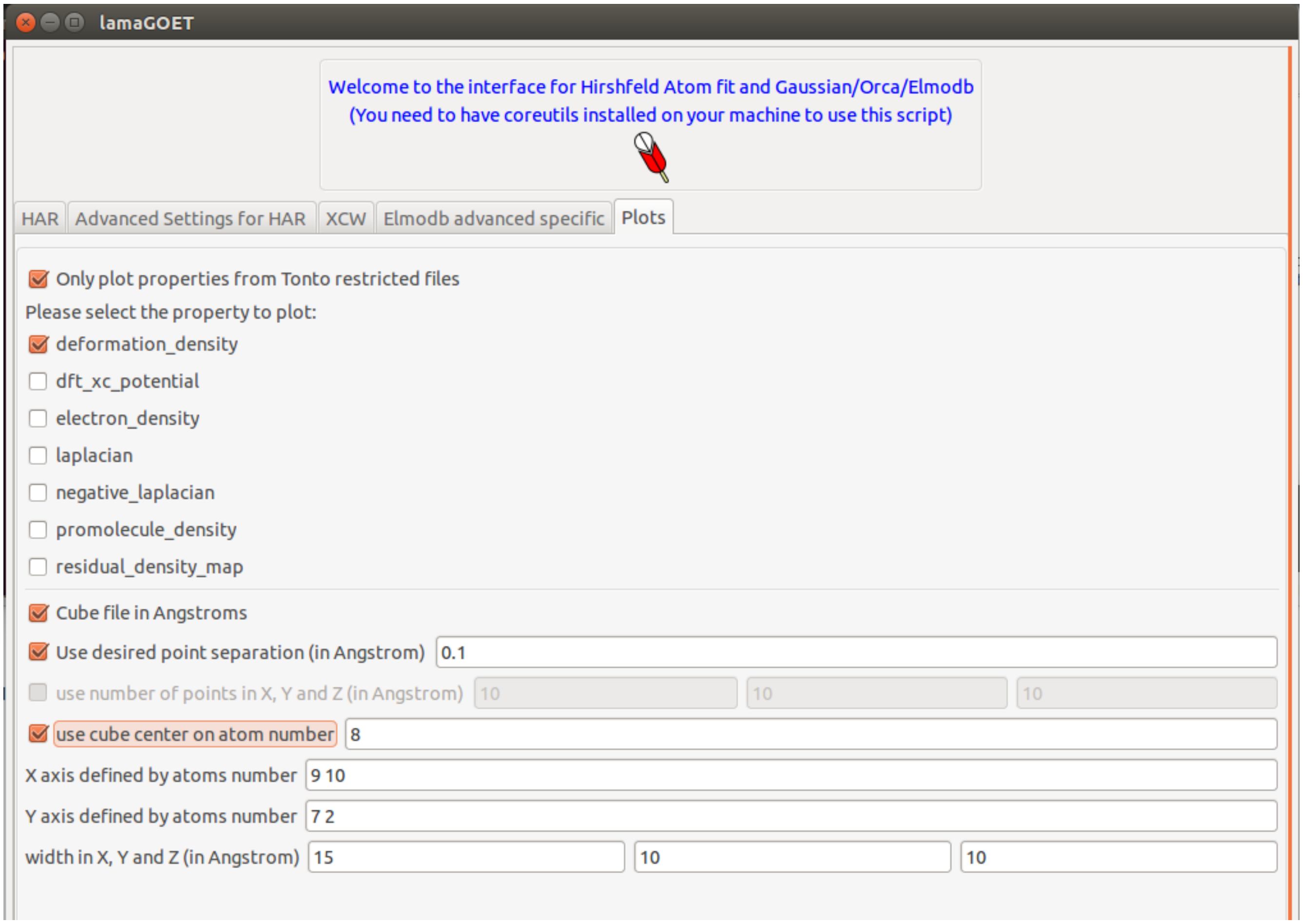

Figure S9. *lamaGOET* GUI showing the input to generate a cube file using a size and origin different from the unit cell.

3 Theoretical optimizations using self consistent cluster charges - SCCC opt

By selecting "SC CC opt with Gaussian and Tonto" in the main window, the *lamaGOET* GUI will initially use *Tonto* to read in the CIF and extract the xyz coordinates. It will then write a *Gaussian* input file. Options such as automatic completion of structures with Z'$<$ 1 are still available, as described in 10. Method and basis sets should be selected using the *Gaussian* format. At this point, there are two possibilities:

- If the checkbox (shown in 21) is selected and the radius of the cluster for point charges is entered, then the first *Gaussian* step is a single point calculation to obtain the electron density of the initial geometry. Afterwards, this wavefunction is sent to *Tonto* for the Hirshfeld partitioning and calculation of point charges according to the crystallographic symmetry. Subsequently, the calculated point charges are used in the next *Gaussian* job which is a geometry optimization. After the first optimization converges, the new geometry is sent back to *Tonto* for re-computation of new cluster charges consistent with the new geometry. The new charges are sent again to a new optimization and these steps are automatically repeated until convergence in energy is achieved. The adopted convergence criterion corresponds to the one provided in the second tab of the *lamaGOET* GUI and, therefore, it should be changed from 0.01 to a reasonable value for the energy difference between the wavefunction obtained from two consecutive calculations. Once convergence is achieved, a frequency calculation is performed as final step. Overall, this procedure could be understood as a quick approximation of a periodic boundary calculation.
- If the checkbox shown in 21 is not selected, *lamaGOET* will initiate only one step including the optimization and frequency calculation of the isolated molecule in *Gaussian* without the use of cluster charges. In this case, *lamaGOET* is simply a handy tool to extract coordinates from a CIF and setup *Gaussian* calculations automatically.

To illustrate the consequences of including point charges in theoretical optimization procedures, we present here different geometry optimizations of the previously reported structure of 9-hydroxy-4-oxocyclopenta[1,2-b]naphthalene-1-carboxaldehyde [6]. This compound can exist in two different tautomeric forms that are energetically similar, where the tautomerization occurs via proton transfer inside an intramolecular hydrogen bond (Figure S10). In the experimental crystal structure refined with HAR, the proton in question (H9) is located more closely to oxygen atom O9 than to O10 (for experimental bond distances see the caption of Figure S10) [6]. We have shown that this preference is triggered by the

asymmetry in the intermolecular interactions of O9 and O10, with O10 being involved in much stronger hydrogen bonds [6].

For the isolated state, at very low levels of theory (HF/STO-3G and HF/def2-SVP), there is only one minimum geometry with the hydrogen atom being closer either to O9 or to O10. For higher levels of theory (B3LYP/cc-pVTZ and B3LYP/6-311++G(2df,p)), there are two minima in a double-well potential which are about 2.5 kJ/mol apart (the geometry with H9 bonded to O9 is energetically lower, the activation energy barrier is about 5.5 kJ/mol). The minimum structures at B3LYP/cc-pVTZ and B3LYP/6-311++G(2df,p) levels in the isolated state agree with the tautomer found in the solid state (for experimental bond distances see caption of Figure S10). If geometry optimizations under periodic boundary conditions are too demanding for a system of interest, an explicit or implicit simulation of the environment is the alternative approach, with the environment being produced by the crystal packing and its symmetry [7]. Here, self-consistent Hirshfeld charges available via the Hirshfeld atom partitioning of quantum-mechanical electron densities in *Tonto* are taken by *lamaGOET* from *Tonto* and placed at symmetry-generated positions to produce a *Gaussian* input file that includes the simulated crystal field.

In this study, we only want to make a methodological observation and refer to the low-level-of-theory calculations. Figure S10 shows that, in the HF/STO-3G case, the correct hydrogen atom position can only be reproduced if the crystal field is accounted for [a) vs. b)], whereas, in the HF/def2-SVP case, the basis set is already sufficient to always reproduce the correct position with H9 bonded to O9 [c) vs. d)]. In turn, now the crystal field has a negligible influence on the hydrogen atom position. In a forthcoming study, we will investigate the quantum-crystallographic electric field and how it influences the position of hydrogen atoms in strong hydrogen bonds. Here, we show that the program *lamaGOET* facilitates the optimization of molecular geometries within the fields of symmetry-generated crystallographic self-consistent cluster charges.

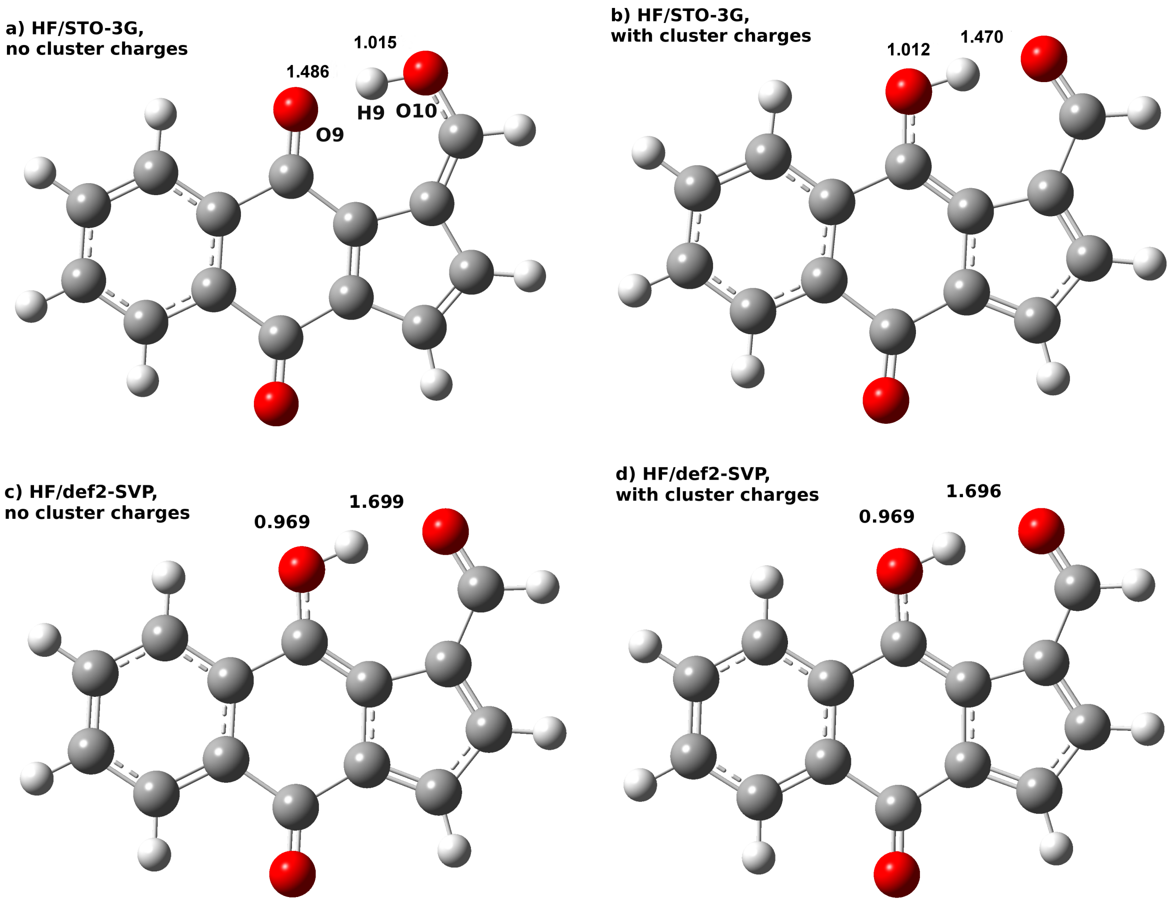

Figure S10. Geometry optimizations of 9-hydroxy-4-oxocyclopenta[1,2-b]naphthalene-1-carbox-aldehyde with O-H bond distances in Å.

First column [a) and c)]: isolated-molecule optimizations. Second column [b) and d)]: optimizations inside a surrounding cluster of point charges within a radius of 8 Å from the central molecule reflecting the crystal symmetry. The point charges are calculated with *Tonto* on the basis of the Hirshfeld atom partitioning scheme, extracted from *Tonto* and placed by *lamaGOET* into a *Gaussian* input file.

First row [a) and b)]: level of theory HF/STO-3G. Second row [c) and d)]: level of theory HF/def2-SVP.

Reported experimental distances in the crystal are d(O9-H9) = 1.15(4) Å and d(O10-H9) = 1.35(4) Å [6].

Graphics produced with the software GaussView [8].

References

1. S. Grimme, S. Ehrlich and L. Goerigk, *J. Comput. Chem.*, 2011, **32**, 1456–1465.
2. M. Douglas and N. M. Kroll, *Ann. Phys.*, 1974, **82**, 89–155.
3. B. A. Hess, *Phys. Rev. A*, 1985, **32**, 756–763.
4. A. Hald, *Int. Stat. Rev.*, 2000, **68**, 137–153.
5. A. D. Becke, *J. Chem. Phys.*, 1988, **88**, 2547–2553.
6. L. A. Malaspina, A. H. White, D. Wege, M. B. Tolmie, B. W. Skelton and S. Grabowsky, *Struct. Chem.*, 2017, **28**, 1343–1357.
7. B. Dittrich, S. Pfitzenreuter and C. B. Hübschle, *Acta Cryst. A*, 2012, **68**, 110–116.
8. R. D. Dennington, T. A. Keith and J. M. Millam, *GaussView 5.0., Gaussian Inc*, 2008.
