# Supplementary material for: lamaGOET: an interface for quantum crystallography
Source: J Appl Crystallogr. 2021 Apr 16;54(Pt 3):987–95. doi: 10.1107/S1600576721002545 (PMC8202027; doi:10.1107/S1600576721002545)

checkCIF/PLATON report

Structure factors have been supplied for datablock(s) gly-l-thr_HAR-ELMO-XCW

THIS REPORT IS FOR GUIDANCE ONLY. IF USED AS PART OF A REVIEW PROCEDURE FOR PUBLICATION, IT SHOULD NOT REPLACE THE EXPERTISE OF AN EXPERIENCED CRYSTALLOGRAPHIC REFEREE.

No syntax errors found. CIF dictionary Interpreting this report

Datablock: gly-l-thr_HAR-ELMO-XCW

Bond precision: C-C = 0.0009 A Wavelength=0.71073

Cell:	a=9.572(3)	b=10.039(3)	c=10.548(3)
	alpha=90	beta=90	gamma=90
Temperature:	110 K		

	Calculated	Reported
Volume	1013.6(5)	1013.6(5)
Space group	P 21 21 21	P 21 21 21
Hall group	P 2ac 2ab	P 2ac 2ab
Moiety formula	C6 H12 N2 O4, 2(H2 O)	C6 H16 N2 O6
Sum formula	C6 H16 N2 O6	C6 H16 N2 O6
Mr	212.21	212.20
Dx,g cm-3	1.391	1.390
Z	4	4
Mu (mm-1)	0.124	0.124
F000	456.0	456.0
F000’	456.30	
h,k,lmax	22,23,24	22,23,24
Nref	12963[7096]	5417
Tmin,Tmax	0.963,0.982	
Tmin’	0.963	

Correction method= Not given

Data completeness= 0.76/0.42 Theta(max)= 54.910

R(reflections)= 0.0265(4579) wR2(reflections)= wR= 0.0216(4579)

S = 0.799 Npar= 271

The following ALERTS were generated. Each ALERT has the format
test-name_ALERT_alert-type_alert-level.
Click on the hyperlinks for more details of the test.

Alert level A

```
PLAT992_ALERT_5_A Repd & Actual _reflns_number_gt Values Differ by        267 Check
```

Author Response: Refinement performed against F using cutoff criterion of 3. The numbers differ due to different formula.

Alert level C

```
PLAT351_ALERT_3_C Long   C-H (X0.96,N1.08A)  C1A      - H1A2     .       1.13 Ang.
PLAT351_ALERT_3_C Long   C-H (X0.96,N1.08A)  C2A      - H2A      .       1.12 Ang.
PLAT353_ALERT_3_C Long   N-H (N0.87,N1.01A)  N2       - H2N      .       1.03 Ang.
PLAT790_ALERT_4_C Centre of Gravity not Within Unit Cell: Resd.  #          1 Note
              C6 H12 N2 O4
PLAT975_ALERT_2_C Check Calcd Resid. Dens.  0.53A   From O2W             0.46 eA-3
PLAT975_ALERT_2_C Check Calcd Resid. Dens.  0.57A   From O2W             0.46 eA-3
```

Alert level G

```
PLAT042_ALERT_1_G Calc. and Reported MoietyFormula Strings  Differ     Please Check
PLAT720_ALERT_4_G Number of Unusual/Non-Standard Labels ..........         12 Note
PLAT790_ALERT_4_G Centre of Gravity not Within Unit Cell: Resd.  #          2 Note
              H2 O
PLAT791_ALERT_4_G Model has Chirality at C2A         (Sohnke SpGr)          S Verify
PLAT791_ALERT_4_G Model has Chirality at C2B         (Sohnke SpGr)          R Verify
PLAT808_ALERT_5_G No Parseable SHELXL Style Weighting Scheme Found     Please Check
PLAT882_ALERT_1_G No Datum for _diffrn_reflns_av_unetI/netI ......     Please Do !
PLAT883_ALERT_1_G No Info/Value for _atom_sites_solution_primary .     Please Do !
PLAT911_ALERT_3_G Missing FCF Refl Between Thmin & STh/L=    0.600         41 Report
PLAT912_ALERT_4_G Missing # of FCF Reflections Above STh/L=  0.600       2467 Note
PLAT913_ALERT_3_G Missing # of Very Strong Reflections in FCF ....          3 Note
PLAT929_ALERT_5_G No Weight Pars,Obs and Calc R1,wR2,S not Checked          ! Info
PLAT955_ALERT_1_G Reported (CIF) and Actual (FCF) Lmax Differ by .          1 Units
PLAT961_ALERT_5_G Dataset Contains no Negative Intensities .......     Please Check
PLAT978_ALERT_2_G Number C-C Bonds with Positive Residual Density.          2 Info
PLAT981_ALERT_1_G No non-zero f" Anomalous Scattering Values Found     Please Check
PLAT986_ALERT_1_G No non-zero f' Anomalous Scattering Values Found     Please Check
```

---

1 **ALERT level A** = Most likely a serious problem - resolve or explain
0 **ALERT level B** = A potentially serious problem, consider carefully
6 **ALERT level C** = Check. Ensure it is not caused by an omission or oversight
17 **ALERT level G** = General information/check it is not something unexpected

6 ALERT type 1 CIF construction/syntax error, inconsistent or missing data
3 ALERT type 2 Indicator that the structure model may be wrong or deficient
5 ALERT type 3 Indicator that the structure quality may be low
6 ALERT type 4 Improvement, methodology, query or suggestion
4 ALERT type 5 Informative message, check

It is advisable to attempt to resolve as many as possible of the alerts in all categories. Often the minor alerts point to easily fixed oversights, errors and omissions in your CIF or refinement strategy, so attention to these fine details can be worthwhile. In order to resolve some of the more serious problems it may be necessary to carry out additional measurements or structure refinements. However, the purpose of your study may justify the reported deviations and the more serious of these should normally be commented upon in the discussion or experimental section of a paper or in the "special_details" fields of the CIF. checkCIF was carefully designed to identify outliers and unusual parameters, but every test has its limitations and alerts that are not important in a particular case may appear. Conversely, the absence of alerts does not guarantee there are no aspects of the results needing attention. It is up to the individual to critically assess their own results and, if necessary, seek expert advice.

Publication of your CIF in IUCr journals

A basic structural check has been run on your CIF. These basic checks will be run on all CIFs submitted for publication in IUCr journals (*Acta Crystallographica*, *Journal of Applied Crystallography*, *Journal of Synchrotron Radiation*); however, if you intend to submit to *Acta Crystallographica Section C* or *E* or *IUCrData*, you should make sure that full publication checks are run on the final version of your CIF prior to submission.

Publication of your CIF in other journals

Please refer to the *Notes for Authors* of the relevant journal for any special instructions relating to CIF submission.

PLATON version of 05/12/2020; check.def file version of 05/12/2020

Datablock gly-l-thr_HAR-ELMO-XCW - ellipsoid plot

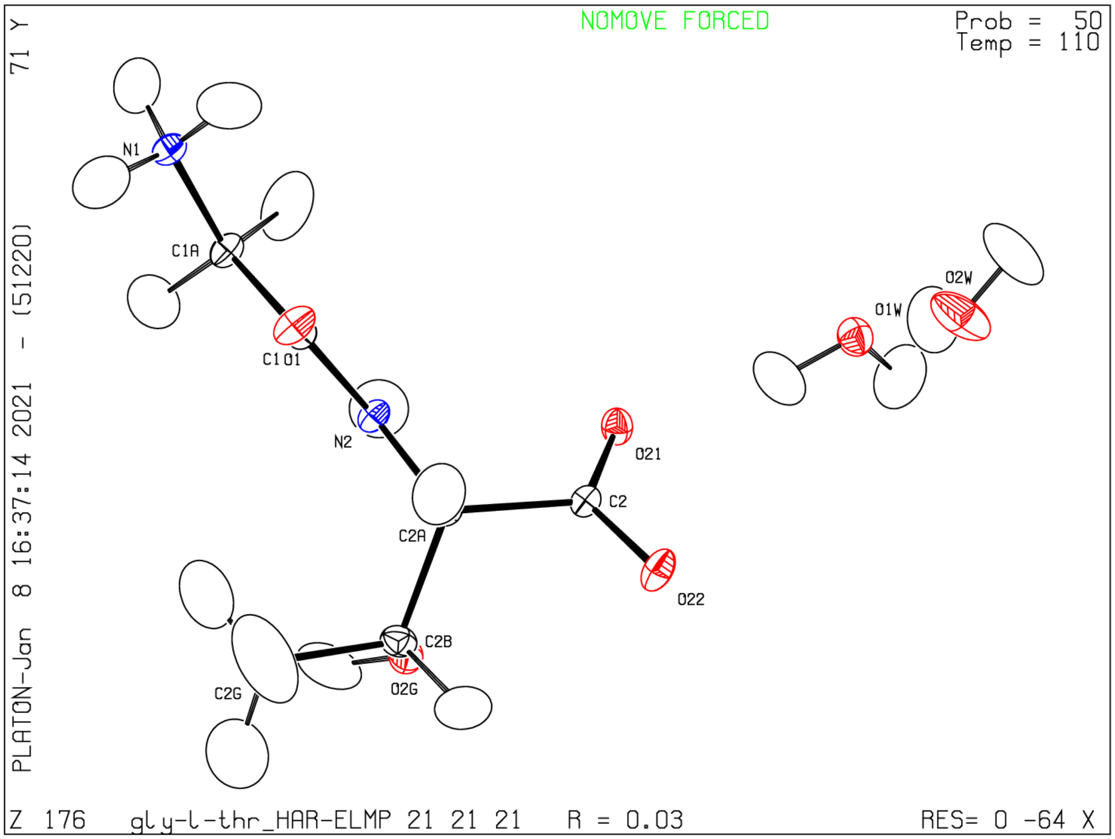

Supplement: Supplementary file 4 [file Gly-L-Thr_HAR-ELMO-XCW_lambda3_checkcif.pdf › Gly-L-Thr_HAR-ELMO-XCW_lambda3_checkcif.pdf]
