# Supplementary material for: lamaGOET: an interface for quantum crystallography
Source: J Appl Crystallogr. 2021 Apr 16;54(Pt 3):987–95. doi: 10.1107/S1600576721002545 (PMC8202027; doi:10.1107/S1600576721002545)

checkCIF/PLATON report

Structure factors have been supplied for datablock(s) nh3_HAR

THIS REPORT IS FOR GUIDANCE ONLY. IF USED AS PART OF A REVIEW PROCEDURE FOR PUBLICATION, IT SHOULD NOT REPLACE THE EXPERTISE OF AN EXPERIENCED CRYSTALLOGRAPHIC REFEREE.

No syntax errors found. CIF dictionary Interpreting this report

Datablock: nh3_HAR

```
Bond precision:  = 0.0000 A              Wavelength=0.71073

Cell:               a=5.1305(8)      b=5.1305(8)      c=5.1305(8)
                    alpha=90         beta=90          gamma=90
Temperature:        160 K

                 Calculated                 Reported
Volume           135.05(6)                  135.05(4)
Space group      P 21 3                     P 21 3
Hall group       P 2ac 2ab 3                P 2ac 2ab 3
Moiety formula   H3 N                       H3 N
Sum formula      H3 N                       H3 N
Mr               17.03                      17.03
Dx,g cm-3        0.838                      0.838
Z                4                          4
Mu (mm-1)        0.064                      0.064
F000             40.0                       40.0
F000'            40.00
h,k,lmax         7,7,7                      7,7,7
Nref             134[ 85]                   134
Tmin,Tmax
Tmin'

Correction method= Not given

Data completeness= 1.58/1.00      Theta(max)= 29.647

R(reflections)= 0.0073( 98)       wR2(reflections)= wR= 0.0064( 98)

S = 1.402                   Npar= 13
```

The following ALERTS were generated. Each ALERT has the format
test-name_ALERT_alert-type_alert-level.
Click on the hyperlinks for more details of the test.

Alert level A

```
PLAT089_ALERT_3_A Poor Data / Parameter Ratio (Zmax < 18) ........       3.77 Note
```

Author Response: Data set has been merged and pruned of systematic absences for HAR.

Alert level B

```
PLAT049_ALERT_1_B Calculated Density Less Than 1.0 gcm-3 .........     0.8378 Check
```

Author Response: This is a re-refinement of previously reported structure ICSD code 84461.

Alert level C

```
REFLE01_ALERT_3_C  The _reflns_threshold_multiplier given is >= 4
            Premultiplier =    4.01
```

Alert level G

```
PLAT019_ALERT_1_G _diffrn_measured_fraction_theta_full/*_max < 1.0      0.884 Report
PLAT152_ALERT_1_G The Supplied and Calc. Volume s.u. Differ by ...          2 Units
PLAT808_ALERT_5_G No Parseable SHELXL Style Weighting Scheme Found     Please Check
PLAT882_ALERT_1_G No Datum for _diffrn_reflns_av_unetI/netI ......     Please Do !
PLAT883_ALERT_1_G No Info/Value for _atom_sites_solution_primary .     Please Do !
PLAT910_ALERT_3_G Missing # of FCF Reflection(s) Below Theta(Min).          1 Note
PLAT911_ALERT_3_G Missing FCF Refl Between Thmin & STh/L=    0.600         26 Report
PLAT912_ALERT_4_G Missing # of FCF Reflections Above STh/L=  0.600          9 Note
PLAT929_ALERT_5_G No Weight Pars,Obs and Calc R1,wR2,S not Checked          ! Info
PLAT955_ALERT_1_G Reported (CIF) and Actual (FCF) Lmax Differ by .          1 Units
PLAT961_ALERT_5_G Dataset Contains no Negative Intensities .......     Please Check
PLAT981_ALERT_1_G No non-zero f" Anomalous Scattering Values Found     Please Check
PLAT986_ALERT_1_G No non-zero f' Anomalous Scattering Values Found     Please Check
```

```
   1 ALERT level A = Most likely a serious problem - resolve or explain
   1 ALERT level B = A potentially serious problem, consider carefully
   1 ALERT level C = Check. Ensure it is not caused by an omission or oversight
  13 ALERT level G = General information/check it is not something unexpected

   8 ALERT type 1 CIF construction/syntax error, inconsistent or missing data
   0 ALERT type 2 Indicator that the structure model may be wrong or deficient
   4 ALERT type 3 Indicator that the structure quality may be low
   1 ALERT type 4 Improvement, methodology, query or suggestion
   3 ALERT type 5 Informative message, check
```

It is advisable to attempt to resolve as many as possible of the alerts in all categories. Often the minor alerts point to easily fixed oversights, errors and omissions in your CIF or refinement strategy, so attention to these fine details can be worthwhile. In order to resolve some of the more serious problems it may be necessary to carry out additional measurements or structure refinements. However, the purpose of your study may justify the reported deviations and the more serious of these should normally be commented upon in the discussion or experimental section of a paper or in the "special_details" fields of the CIF. checkCIF was carefully designed to identify outliers and unusual parameters, but every test has its limitations and alerts that are not important in a particular case may appear. Conversely, the absence of alerts does not guarantee there are no aspects of the results needing attention. It is up to the individual to critically assess their own results and, if necessary, seek expert advice.

Publication of your CIF in IUCr journals

A basic structural check has been run on your CIF. These basic checks will be run on all CIFs submitted for publication in IUCr journals (*Acta Crystallographica*, *Journal of Applied Crystallography*, *Journal of Synchrotron Radiation*); however, if you intend to submit to *Acta Crystallographica Section C* or *E* or *IUCrData*, you should make sure that full publication checks are run on the final version of your CIF prior to submission.

Publication of your CIF in other journals

Please refer to the *Notes for Authors* of the relevant journal for any special instructions relating to CIF submission.

PLATON version of 22/12/2019; check.def file version of 13/12/2019

Datablock nh3_HAR - ellipsoid plot

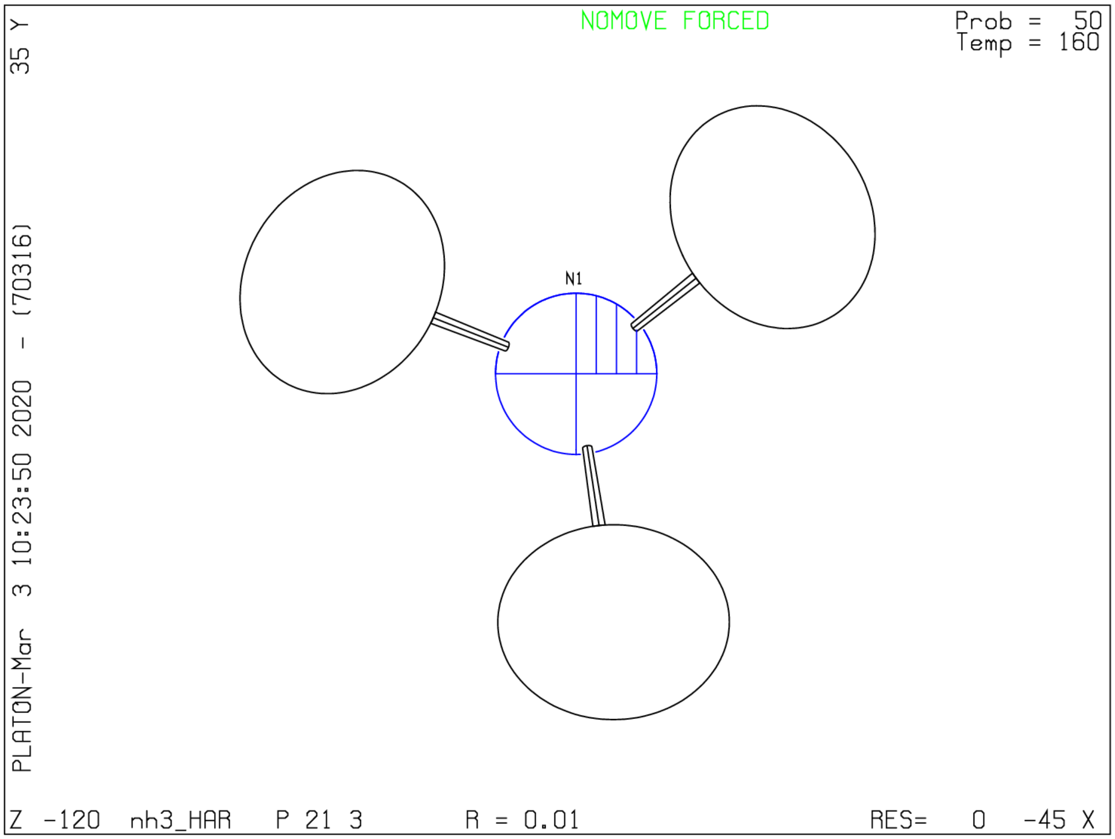

Supplement: Supplementary file 4 [file nh3_checkcif.pdf › nh3_checkcif.pdf]
